# Supplementary material for: What effect might border screening have on preventing importation of COVID-19 compared with other infections?: considering the additional effect of post-arrival isolation
Source: Epidemiol Infect. 2022 Aug 11;150:e159. doi: 10.1017/S0950268822001327 (PMC9464505; doi:10.1017/S0950268822001327)
Supplement: Supplementary file 1 [file hygsup.zip › S0950268822001327sup001.pdf]

Impact of Double Screening Travellers for Varying Diseases

This notebook is to be used to obtain detection rates from the updated BoarderScreening code contained in the SIRA package. As such, this work, as opposed to that contained in the `BorderScreening` notebook, looks to take advantage of the newly added functionaltiy that allows the user to model all incoming travellers undertaking a period of additional isolation after arrival into the destination country. The model then reports the proportion of infected travellers (those that successfully board their flight) that will be detected by testing adminisitered either on arrival, or at the end of their self-isolation period. We then plan to report the data obtained by this updated model in paper which is to follow up the original border screening article. In this new paper, we plan to compare the results of the two models and determine what affect requiring all incoming travellers to complete a period of self-isolation has on detection rates during a disease outbreak outbreaks.

Aside from assessing a range of diseases, we shall also be varying flight time to model situations where the outbreak has occured in countries of varying distance away from the destination country.

In [1]:
```
from sira import border_screening
import matplotlib.pyplot as plt
import numpy.random as rand
import scipy.stats as stats
import matplotlib as mpl
import pandas as pd
import numpy as np
import os
```

In [2]:
```
flight_ranges = [(3,5), (7,9), (11,13)]

exp_ranges = []

diseases =['Ebola', 'SARS', 'Influenza']

FULL_diseases =['COVID-19', 'Influenza', 'SARS', 'Ebola']

inc_dists = {'Ebola':stats.gamma, 'SARS':stats.weibull_min, 'Influenza':stats.gamma,
             'COVID-19': stats.lognorm}

inc_params = {'Ebola':{'a':8.27054, 'scale': 1.51139},
              'SARS':{'c':2.59, 'scale':5.8},
              'Influenza':{'a':4.7556, 'scale':0.3007},
              'COVID-19': {'s':0.47238, 'scale': np.exp(1.6112)}}
```

Incubation period distributions

We now quickly test that these distributions look as they should. We therefore quickly draw the dstributions described above to see how they appear.

In [3]:

```
dists_graphs = []
time_range = {'Ebola': np.linspace(0, 30, 10000), 'SARS': np.linspace(0, 20, 10000), 'Influenza':
              'COVID-19': np.linspace(0, 20, 10000)}

fig, (ax1, ax2, ax3, ax4) = plt.subplots(4,1)
fig.set_size_inches(10,10)
fig.tight_layout(pad=3.0)

ax = {'Ebola': ax1, 'SARS': ax2, 'Influenza': ax3, 'COVID-19': ax4}
fig.suptitle('Ebola, SARS, Influenza and COVID-19 incubation dsitributions', y =1.02)

for dis in diseases:
    ax[dis].plot(time_range[dis], inc_dists[dis].pdf(time_range[dis], **inc_params[dis]))
    ax[dis].set_title('{} incubation period distribution (days)'.format(dis))

plt.show()
```

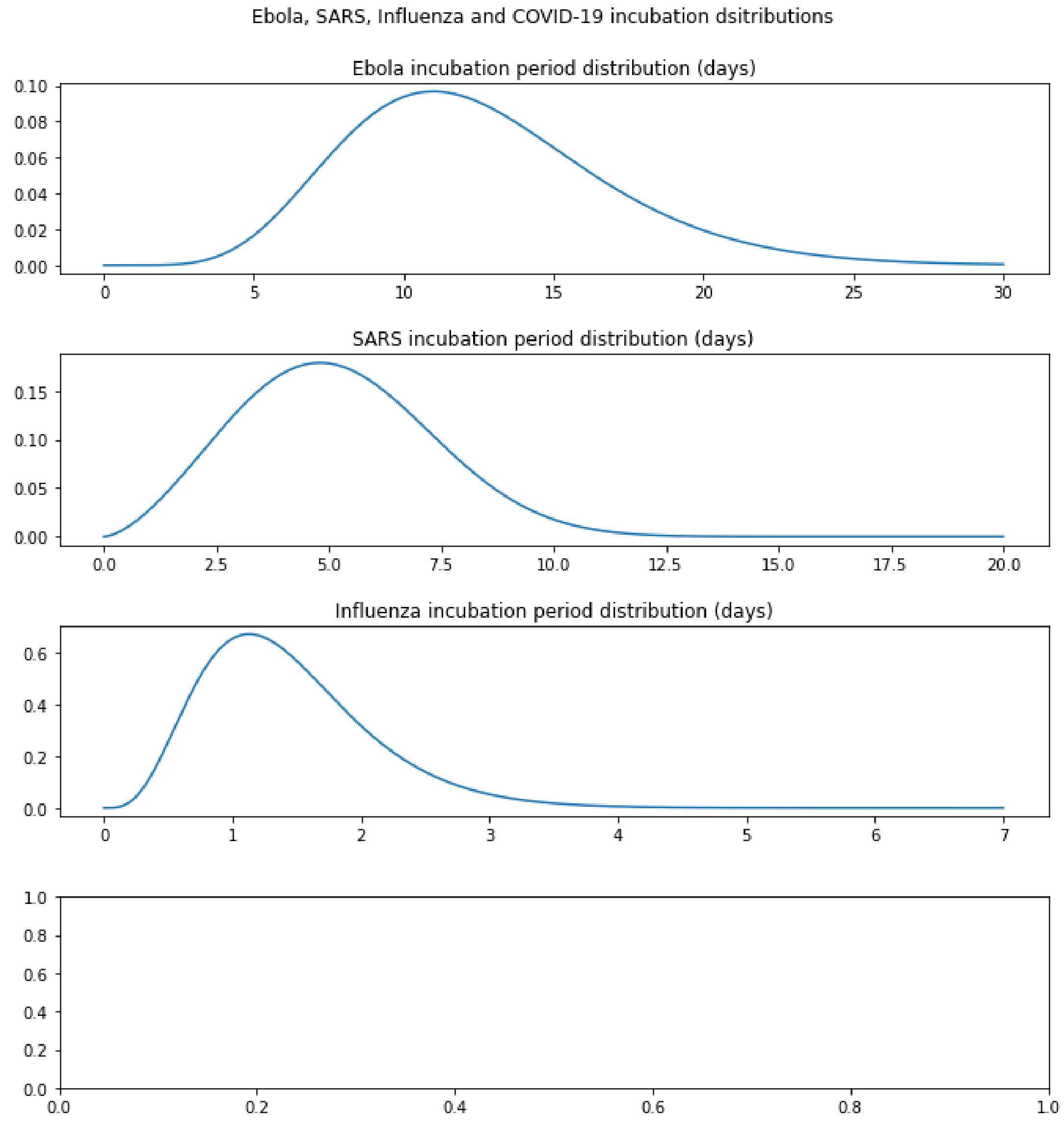

In [4]:

```
# Values to describe the upper limit to the windows in which travellers have been infected prior
# flights

border_shut = [72, 168, 336]

# Values to describe the different times that travellers will be required to isolate for in diffe
isolation_times = [i*24 for i in range(1, 15)]
```

In [5]:

```
df = pd.DataFrame(columns=['Disease name',
                           'Flight time range',
                           'Exposure range (days)',
                           'Self-isolation period (days)',
                           'Calculated border screening success rate'], index=range(1,600))
```

In [6]:

```
for i in range(126):
    fly = flight_ranges[i % 3]
    exp = border_shut[(int(np.floor(i / 3)) % 3)]
    iso = isolation_times[int(np.floor(i/9))]
    tmp_list = []

    for _ in range(100):
        screening = border_screening(num_people = 10000,
                                     flight_dist = rand.uniform,
                                     exp_dist = rand.uniform,
                                     inc_dist = rand.gamma,
                                     time_scale = 'days',
                                     second_screen = iso,
                                     release_time = iso+0.0001,
                                     **{
                                        'exp':{'low':0, 'high':exp},
                                        'inc':{'shape':8.27054, 'scale':1.51139},
                                        'flight':{'low':fly[0], 'high':fly[1]}
                                        }
                                     )
        tmp_list.append(screening)

    avg_screening = np.mean(tmp_list)
    percentile_5 = np.percentile(tmp_list, 5)
    percentile_95 = np.percentile(tmp_list, 95)

    screening_rate = f'{avg_screening} ({percentile_5} - {percentile_95})'

    df.loc[i+1] = pd.Series({'Disease name': 'Ebola',
                             'Exposure range (days)': '(0, {})'.format(exp/24),
                             'Flight time range': 'Uniform {}'.format(fly),
                             'Self-isolation period (days)': iso/24,
                             'Calculated border screening success rate': screening_rate
```

In [7]:

```
for i in range(126):
    fly = flight_ranges[i % 3]
    exp = border_shut[(int(np.floor(i / 3)) % 3)]
    iso = isolation_times[int(np.floor(i/9))]
    tmp_list = []

    for _ in range(100):
        screening = border_screening(num_people = 10000,
                                     flight_dist = rand.uniform,
                                     exp_dist = rand.uniform,
                                     inc_dist = rand.weibull,
                                     time_scale = 'days',
                                     second_screen = iso,
                                     release_time = iso+0.0001,
                                     **{
                                        'exp':{'low':0, 'high':exp},
                                        'inc':{'c':2.59, 'scale':5.8},
                                        'flight':{'low':fly[0], 'high':fly[1]}
                                        }
                                     )

        tmp_list.append(screening)

    avg_screening = np.mean(tmp_list)
    percentile_5 = np.percentile(tmp_list, 5)
    percentile_95 = np.percentile(tmp_list, 95)

    screening_rate = f'{avg_screening} ({percentile_5} - {percentile_95})'

    df.loc[i+127] = pd.Series({'Disease name':'SARS',
                               'Flight time range': 'Uniform {}'.format(fly),
                               'Exposure range (days)': '(0, {})'.format(exp/24),
                               'Self-isolation period (days)': iso/24,
                               'Calculated border screening success rate': screening_rate})
```

In [8]:

```
for i in range(126):
    fly = flight_ranges[i % 3]
    exp = border_shut[(int(np.floor(i / 3)) % 3)]
    iso = isolation_times[int(np.floor(i/9))]
    tmp_list = []

    for _ in range(100):
        screening = border_screening(num_people = 10000,
                                     flight_dist = rand.uniform,
                                     exp_dist = rand.uniform,
                                     inc_dist = rand.gamma,
                                     time_scale = 'days',
                                     second_screen = iso,
                                     release_time = iso+0.0001,
                                     **{
                                        'exp':{'low':0, 'high':exp},
                                        'inc':{'shape':4.7556, 'scale':0.3007},
                                        'flight':{'low':fly[0], 'high':fly[1]}
                                        }
                                     )
        tmp_list.append(screening)

    avg_screening = np.mean(tmp_list)
    percentile_5 = np.percentile(tmp_list, 5)
    percentile_95 = np.percentile(tmp_list, 95)

    screening_rate = f'{avg_screening} ({percentile_5} - {percentile_95})'

    df.loc[i+253] = pd.Series({'Disease name':'Influenza',
                               'Flight time range': 'Uniform {}'.format(fly),
                               'Exposure range (days)': '(0, {})'.format(exp/24),
                               'Self-isolation period (days)': iso/24,
                               'Calculated border screening success rate': screening_rate})
```

In [ ]:

```
for i in range(126):
    fly = flight_ranges[i % 3]
    exp = border_shut[(int(np.floor(i / 3)) % 3)]
    iso = isolation_times[int(np.floor(i/9))]
    tmp_list = []

    for _ in range(100):
        screening = border_screening(num_people = 10000,
                                     flight_dist = rand.uniform,
                                     exp_dist = rand.uniform,
                                     inc_dist = rand.lognormal,
                                     time_scale = 'days',
                                     second_screen = iso,
                                     release_time = iso+0.0001,
                                     **{
                                        'exp':{'low':0, 'high':exp},
                                        'inc':{'sigma':0.47238, 'mean': 1.6112},
                                        'flight':{'low':fly[0], 'high':fly[1]}
                                        }
                                     )

        tmp_list.append(screening)

    avg_screening = np.mean(tmp_list)
    percentile_5 = np.percentile(tmp_list, 5)
    percentile_95 = np.percentile(tmp_list, 95)

    screening_rate = f'{avg_screening} ({percentile_5} - {percentile_95})'

    df.loc[i+379] = pd.Series({'Disease name':'COVID-19',
                               'Flight time range': 'Uniform {}'.format(fly),
                               'Exposure range (days)': '(0, {})'.format(exp/24),
                               'Self-isolation period (days)': iso/24,
                               'Calculated border screening success rate': screening_rate})

df = df.dropna().astype({'Self-isolation period (days)': int, 'Calculated border screening succes
```

In [32]:

```
def trim_string(string):
    new_string = ''
    if type(string) == str:
        split_string = string.split(' ')
        for i, splt in enumerate(split_string):
            splt = splt.replace('(', '')
            splt = splt.replace(')', '')
            if splt != '-':
                splt = str(np.round(float(splt), 3))

            if i == 0:
                new_string += splt
            elif i == 1:
                new_string += ' (' + splt
            elif i == 3:
                new_string += ' ' + splt +')'
            else:
                new_string += ' ' + splt
    return new_string
```

In [36]:

```
df2 = df.copy()

df2['Calculated border screening success rate'] = df2['Calculated border screening success rate']
df2.to_csv(save_path)
```

In [34]:

```
df
```

Out[34]:

	Disease name	Flight time range	Exposure range (days)	Self-isolation period (days)	Calculated border screening success rate
**1**	Ebola	Uniform (3, 5)	(0, 3.0)	1.0	0.0009560964214057478 (0.0005000500050005 - 0....
**2**	Ebola	Uniform (7, 9)	(0, 3.0)	1.0	0.0013131074170034513 (0.0008 - 0.001805351569...
**3**	Ebola	Uniform (11, 13)	(0, 3.0)	1.0	0.0017001584308672197 (0.0011002145423584166 -...
**4**	Ebola	Uniform (3, 5)	(0, 7.0)	1.0	0.019407828905979908 (0.01741101758360312 - 0....
**5**	Ebola	Uniform (7, 9)	(0, 7.0)	1.0	0.023183394830144574 (0.020773048792003136 - 0...
**...**	...	...	...	...	...
**595**	NaN	NaN	NaN	NaN	NaN
**596**	NaN	NaN	NaN	NaN	NaN
**597**	NaN	NaN	NaN	NaN	NaN
**598**	NaN	NaN	NaN	NaN	NaN
**599**	NaN	NaN	NaN	NaN	NaN

599 rows × 5 columns

In [10]:

```
cwd_path = os.getcwd()
save_path = os.path.join(cwd_path, 'DoubleScreening_VaryingDisease_2.csv')

df.to_csv(save_path)
```

In [223]:

```
fig, ax = plt.subplots(1,1, figsize=(15,10))
plt.xlabel('xlabel', fontsize=15)
plt.ylabel('Detection rate', fontsize=15)

name = 'COVID-19'
name_subdf = df[df['Disease name'] == name]

for exp, group in name_subdf.groupby('Exposure range (days)'):
    group.groupby('Self-isolation period (days)')['Calculated border screening success rate'].mea
           y='Calculated border screening success rate',
           ax=ax,
           title = 'Detection rates of travellers infected with COVID-19 after undergoing varying
           label = 'Exposure window: {}'.format(exp),
           fontsize=15,
           grid=True)

handles, labels = ax.get_legend_handles_labels()
# sort both labels and handles by labels
labels, handles = zip(*sorted(zip(labels, handles), key=lambda t: float(t[0].split()[-1].strip(')
ax.legend(handles, labels, loc='lower right')
```

Out[223]: <matplotlib.legend.Legend at 0x1b0c8b89ac8>

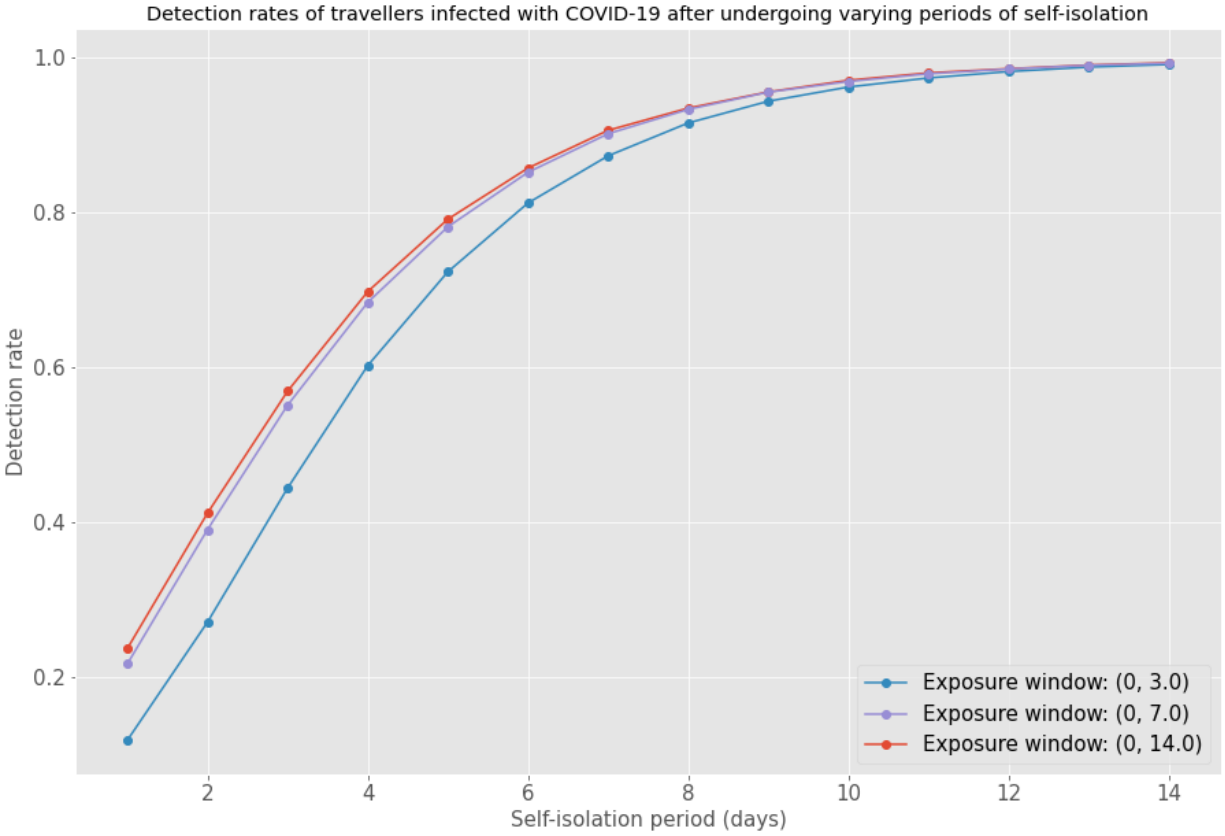

In [231]:

```
fig, (ax1, ax2, ax3) = plt.subplots(3,1, figsize=(15,30))
ax = [ax1, ax2, ax3]
plt.xlabel('xlabel', fontsize=15)
plt.ylabel('Detection rate', fontsize=15)

for i in range(3):
    name = diseases[i]
    name_subdf = df[df['Disease name'] == name]
    for exp, group in name_subdf.groupby('Exposure range (days)'):
        group.groupby('Self-isolation period (days)')['Calculated border screening success rate']
                y='Calculated border screening success rate',
                ax=ax[i],
                title = 'Detection rates of travellers infected with {} after undergoing varying p
                label = 'Exposure window: {}'.format(exp),
                fontsize=15,
                grid=True)

        handles, labels = ax[i].get_legend_handles_labels()
        labels, handles = zip(*sorted(zip(labels, handles), key=lambda t: float(t[0].split()[-1].
        ax[i].legend(handles, labels, loc='lower right')
        ax[i].set_ylabel('Detection rate', fontsize=15)
```

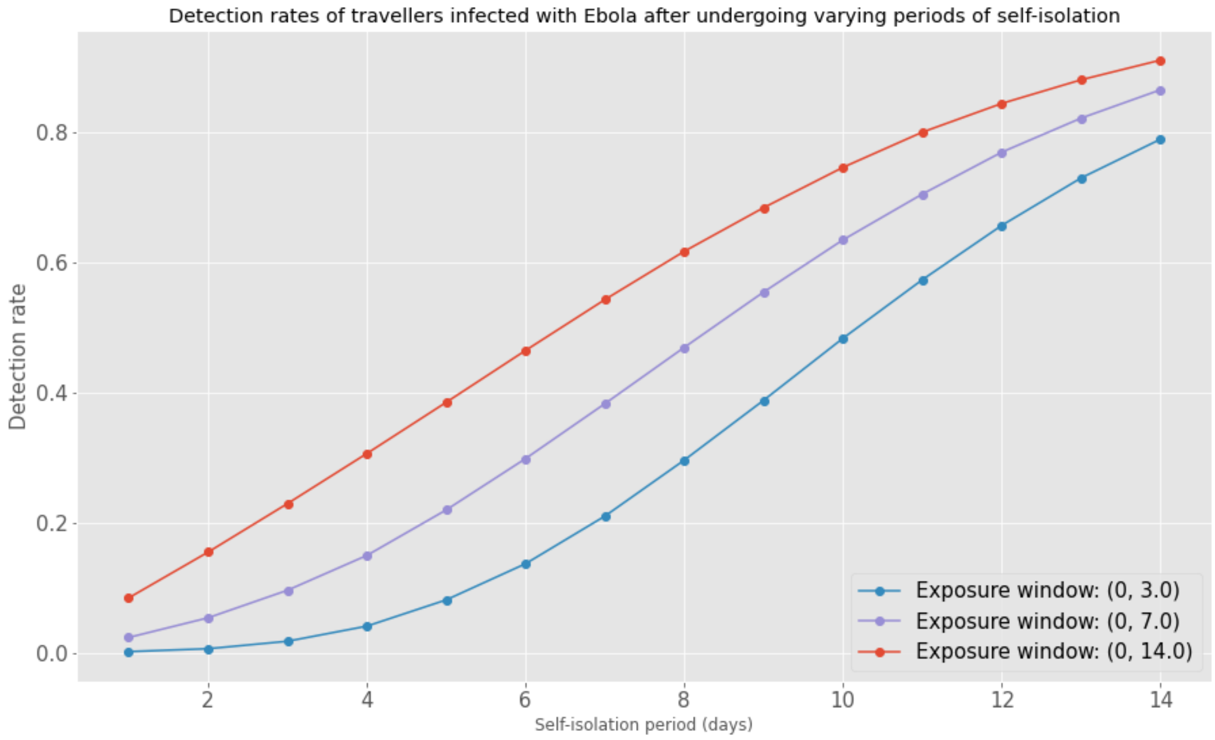

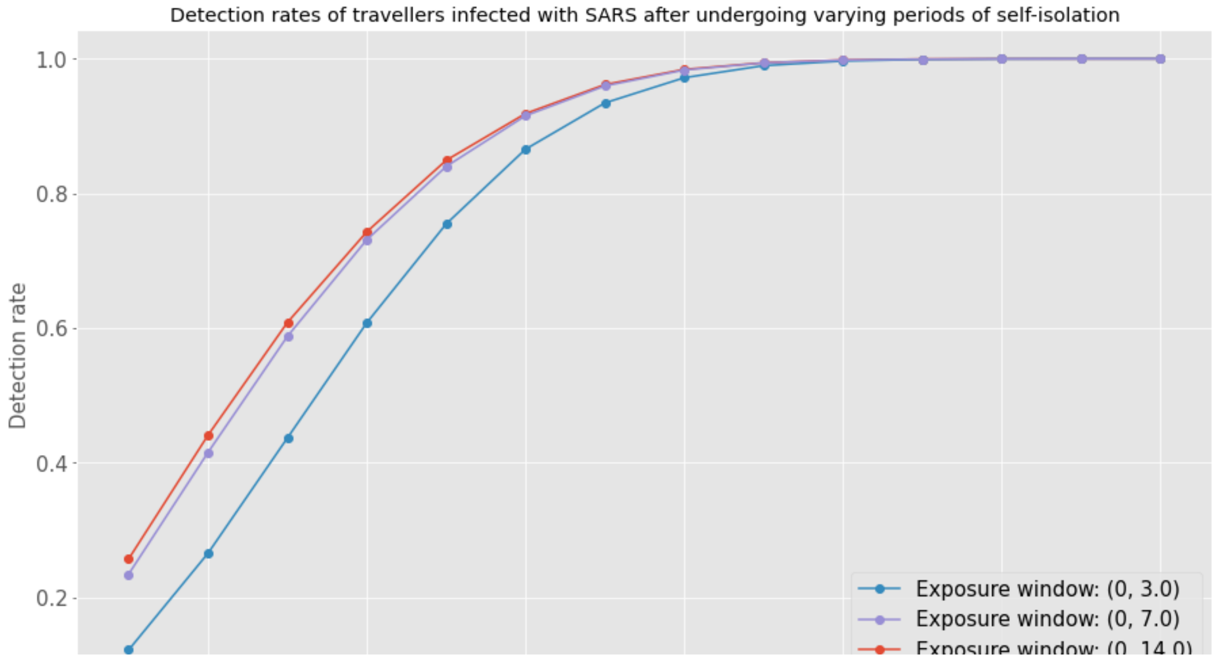

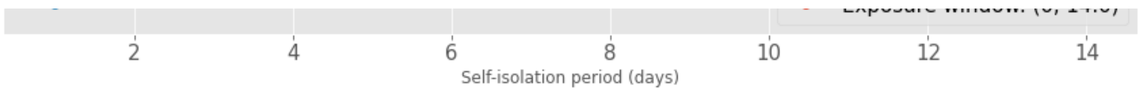
2
4
6
8
10
12
14
Self-isolation period (days)

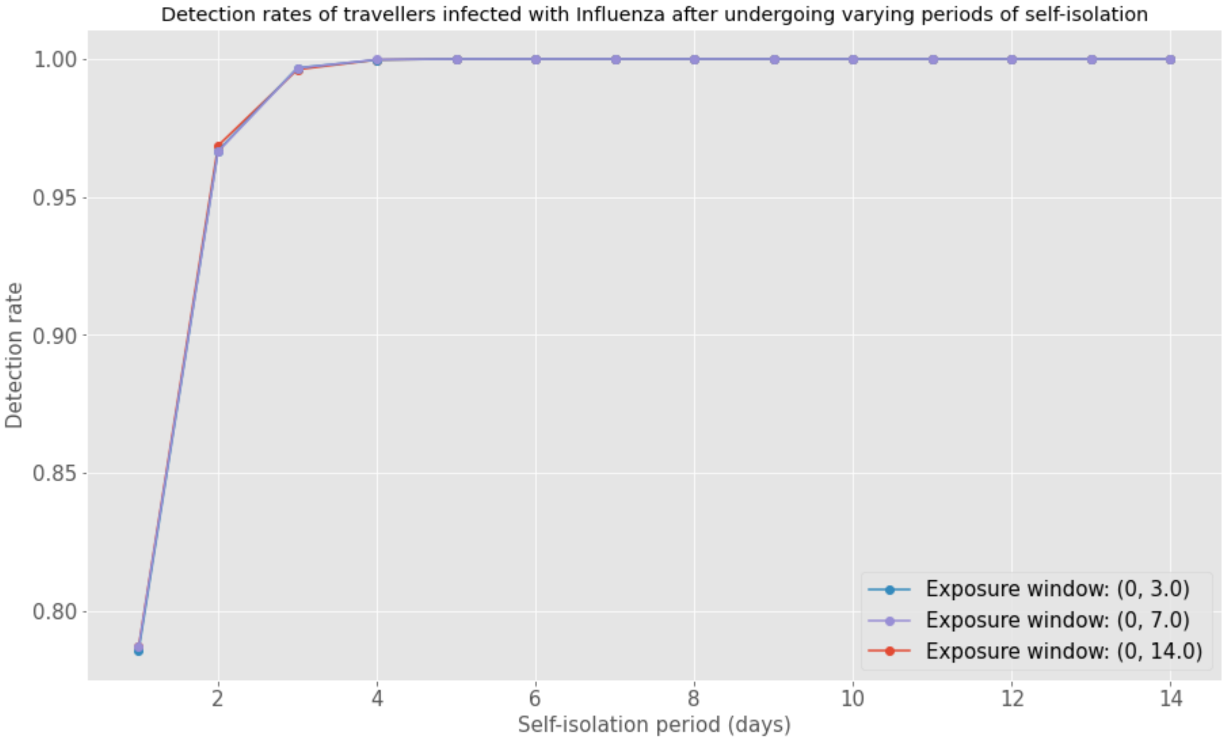
Detection rates of travellers infected with Influenza after undergoing varying periods of self-isolation
Detection rate
1.00
0.95
0.90
0.85
0.80
2
4
6
8
10
12
14
Self-isolation period (days)
Exposure window: (0, 3.0)
Exposure window: (0, 7.0)
Exposure window: (0, 14.0)

In [281]:

```
mpl.rcParams['axes.prop_cycle'] = mpl.cycler(color=["r", "g", "b"])

fig, ((ax1, ax2), (ax3, ax4)) = plt.subplots(2,2, figsize=(18,18))
ax = [ax1, ax2, ax3, ax4]
plt.xlabel('xlabel', fontsize=20)

for i in range(4):
    name = FULL_diseases[i]
    name_subdf = df[df['Disease name'] == name]
    for exp, group in name_subdf.groupby('Exposure range (days)'):
        group.groupby('Self-isolation period (days)')['Calculated border screening success rate']
                 y='Calculated border screening success rate',
                 ax=ax[i],
                 label = 'Exposure window: {}'.format(exp),
                 fontsize=20,
                 #colormap='plasma',
                 grid=True)

        handles, labels = ax[i].get_legend_handles_labels()
        labels, handles = zip(*sorted(zip(labels, handles), key=lambda t: float(t[0].split()[-1].
        #ax[i].legend(handles, labels, loc='lower right')
        ax[i].set_title(f'{name}', fontsize=20)
        if (i % 2) == 0:
            ax[i].set_ylabel('Detection rate', fontsize=20)

        if i in [2,3]:
            ax[i].set_xlabel('Self-isolation period (days)', fontsize=20)
        else:
            ax[i].set_xlabel('', fontsize=20)

fig.legend(handles, labels, loc='lower center', fontsize=20)

fig.savefig('self_isolation_plot.png')
```

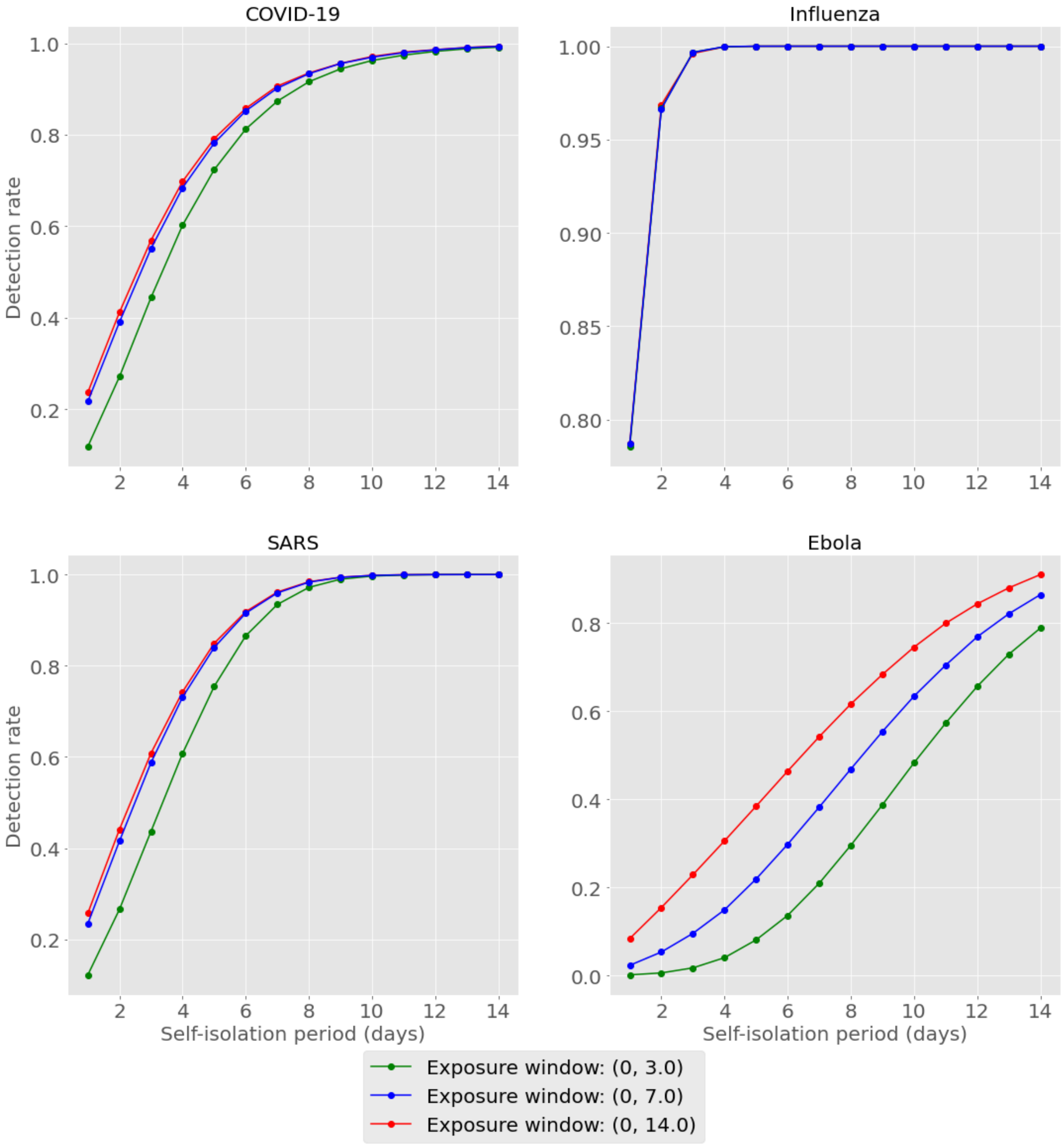

In [271]:

```
df2 = pd.DataFrame(columns=['Disease name',
                            'Exposure window',
                            *list(range(1,15))])
for i in range(4):
    name = FULL_diseases[i]
    name_subdf = df[df['Disease name'] == name]
    for exp, group in name_subdf.groupby('Exposure range (days)'):
        row = group.groupby('Self-isolation period (days)')['Calculated border screening success
        row['Disease name'] = name
        row['Exposure window'] = exp
        df2 = df2.append(row, ignore_index=True)
```

In [273]:

```
df2.to_csv('mean_scr_iso_res.csv')
```
